# Serum Metabolomic Signatures for Knee Cartilage Volume Loss over 10 Years in Community-Dwelling Older Adults

**DOI:** 10.3390/life12060869

**Published:** 2022-06-10

**Authors:** Zikun Xie, Dawn Aitken, Ming Liu, Guanghua Lei, Graeme Jones, Flavia Cicuttini, Guangju Zhai

**Affiliations:** 1 Division of Biomedical Sciences (Genetics), Faculty of Medicine, Memorial University of Newfoundland, St. John’s, NL A1B 3V6, Canada; zikunx@mun.ca (Z.X.); ming.liu@med.mun.ca (M.L.); 2 Xiangya Hospital, Central South University, Changsha 410008, China; lei_guanghua@csu.edu.cn; 3 Menzies Institute for Medical Research, University of Tasmania, Hobart 7005, Australia; dawn.aitken@utas.edu.au (D.A.); graeme.jones@utas.edu.au (G.J.); 4 Department of Epidemiology and Preventive Medicine, Monash University Medical School, Melbourne 3006, Australia; flavia.cicuttini@monash.edu

**Keywords:** knee osteoarthritis, MRI, metabolomics, acylcarnitines

## Abstract

Osteoarthritis (OA) is the most prevalent joint disorder characterized by joint structural pathological changes with the loss of articular cartilage as its hallmark. Tools that can predict cartilage loss would help identify people at high risk, thus preventing OA development. The recent advance of the metabolomics provides a new avenue to systematically investigate metabolic alterations in disease and identify biomarkers for early diagnosis. Using a metabolomics approach, the current study aimed to identify serum metabolomic signatures for predicting knee cartilage volume loss over 10 years in the Tasmania Older Adult Cohort (TASOAC). Cartilage volume was measured in the medial, lateral, and patellar compartments of the knee by MRI at baseline and follow-up. Changes in cartilage volume over 10 years were calculated as percentage change per year. Fasting serum samples collected at 2.6-year follow-up were metabolomically profiled using the TMIC Prime Metabolomics Profiling Assay and pairwise metabolite ratios as the proxies of enzymatic reaction were calculated. Linear regression was used to identify metabolite ratio(s) associated with change in cartilage volume in each of the knee compartments with adjustment for age, sex, and BMI. The significance level was defined at α = 3.0 × 10^−6^ to control multiple testing. A total of 344 participants (51% females) were included in the study. The mean age was 62.83 ± 6.13 years and the mean BMI was 27.48 ± 4.41 kg/m^2^ at baseline. The average follow-up time was 10.84 ± 0.66 years. Cartilage volume was reduced by 1.34 ± 0.72%, 1.06 ± 0.58%, and 0.98 ± 0.46% per year in the medial, lateral, and patellar compartments, respectively. Our data showed that the increased ratios of hexadecenoylcarnitine (C16:1) to tetradecanoylcarnitine (C14) and C16:1 to dodecanoylcarnitine (C12) were associated with 0.12 ± 0.02% reduction per year in patellar cartilage volume (both *p* < 3.03 × 10^−6^). In conclusion, our data suggested that alteration of long chain fatty acid β-oxidation was involved in patellar cartilage loss. While confirmation is needed, the ratios of C16:1 to C14 and C12 might be used to predict long-term cartilage loss.

## 1. Introduction

Osteoarthritis (OA) is the most prevalent joint disorder, affecting approximately 10% of people over 60 years old around the world [1], and resulting in a substantial economic burden on our society, with an aggregate cost of ~2.5% of the gross domestic product [2]. However, there is no cure for it yet and the pathogenesis of OA remains elusive. While it is recognized that all the joint tissues are involved in the development of OA, the articular cartilage loss is its major hallmark [3]. A study showed that knee cartilage volume is lost at a rate of 1.2% per year on average in a general population cohort [4], and could be up to 3.5% in symptomatic knee OA patients [5]. Abnormal cartilage metabolism as a molecular derangement would play a vital role in cartilage volume loss over time. Thus, understanding the alteration of cartilage metabolism and identifying biomarkers associated with cartilage loss could provide new avenues for developing intervention strategies for preventing cartilage loss and OA development.

The recent advance of metabolomics provides a new avenue to systematically investigate metabolic alterations in disease and to identify biomarkers and endotypes for disease stratification, early diagnosis, and treatment monitoring [6,7]. The application of metabolomics to OA research in recent years has produced promising results and identified several metabolic biomarkers and altered metabolic pathways to be involved in OA such as arginine and proline metabolism [8,9,10], the conversion pathway of phosphatidylcholine to lysophosphatidylcholine [11,12], the phenylalanine degradation pathway, and branched-chain amino acid catabolism [5]. Recently, we found that the lysophosphatidylcholine 18:2 (lysoPC 18:2) to phosphatidylcholine 44:3 (PC 44:3) ratio was associated with the knee cartilage volume loss, particularly in the lateral compartment [12]. However, the sample size was small, the study participants were all symptomatic knee OA patients, and the follow-up time was only two years. Thus, we undertook the current study to further investigate the metabolic alteration in knee cartilage volume loss in a population-based cohort with a much longer follow-up time (over 10 years).

## 2. Materials and Methods

### 2.1. Study Participants

The study was part of the Tasmanian Older Adult Cohort Study (TASOAC), which is a prospective, population-based study aimed at identifying the environmental, genetic, and biochemical factors associated with the development and progression of OA [13]. The study was initiated in 2002 and the participants were randomly chosen from the electoral roll (population 229,000) in southern Tasmania, with an equal number of men and women and an age range between 50 to 80 years old. Participants were excluded if they had any implants that would prevent them from undergoing an MRI or they were living in a nursing home. The study was approved by the Southern Tasmanian Health and Medical Human Research Ethics Committee (reference number H0006488), and all participants provided written informed consent.

### 2.2. Demographic Information

Demographic information was collected by a self-administered questionnaire, and anthropometric data including height and weight were measured at clinical interview. Body mass index (BMI) was calculated by dividing weight in kilograms by height in meters squared. Age was calculated at baseline.

### 2.3. MRI and Cartilage Volume Measurement

MRI scans were performed on the right knees of the study participants by using a 1.5T whole body magnetic resonance unit (Picker, Cleveland, OH, USA) and a Siemens apparatus (Espree, Erie, PA, USA) with the following sequences: (i) a T1-weighted fat saturation 3D gradient-recalled acquisition in the steady state; and (ii) a T2-weighted fat saturation 2D fast spin echo. The image sequence is explained elsewhere [14].

The volume of the knee cartilage was measured by a well-trained reader blinded for clinical information on a separate workstation using OsiriX software (University of Geneva, Geneva, Switzerland) [15]. Cartilage volume measurements with the chronological order known for participants who had MRIs at both baseline and 10-year follow-up were conducted with the baseline and 10-year MRIs paired. The volume of each cartilage plate (patella, medial tibia and lateral tibia) was separated by manually drawing the disjoint contour section by section around the cartilage boundary. The coefficient of variation (CV) for cartilage volume measurement was 2.1% for the medial tibia, 2.2% for the lateral tibia, and 2.6% for the patella [15].

### 2.4. Serum Metabolomics Profiling

At the 2.6-year follow-up, serum samples were collected after overnight fasting and stored in a freezer at −80 °C until analysis. Metabolomic profiling was performed using the TMIC Prime Metabolomics Profiling Assay, which measures a total of 143 metabolites including 40 acylcarnitines, 25 amino acids and derivatives, 23 biogenic amines, one amine oxides, one carboxylic acid, one monosaccharide, 17 organic acids, 34 phospho-and sphingolipids, and one vitamin and cofactor. A full list of the metabolites is provided in the Supplementary Table S1. The profiling was done at The Metabolomics Innovation Centre (TMIC) using an AB SCIEX QTRAP^®^4000 mass spectrometer (Sciex Canada, Concord, ON, Canada) equipped with an Agilent 1260 series ultra-high-performance liquid chromatography (UHPLC) system (Agilent Technologies, Palo Alto, CA, USA). The Analyst software 1.6.2 (Concord, ON, Canada) was used to control the entire assay’s workflow and the metabolite concentrations were reported in μM. The CV for the metabolites ranged between 1.16–15.93%.

### 2.5. Statistical Analysis

The cartilage volume changes in the medial, lateral, and patellar compartments of the knee were calculated as percentage change per year which was equal to [100 * (follow-up cartilage volume-baseline cartilage volume)/baseline cartilage volume/time between two scans (in years)] [13]. The metabolomics data went through the following quality control (QC) procedures: metabolites with missing values or concentrations below the limit of detection (LOD) in more than 10% of the samples were removed from subsequent analysis to minimize the false positive results as a standard practice in metabolomics studies [16]. Principal component analysis (PCA) was used to assess batch effects, which showed that there were no batch effects in our experiment; therefore, no batch correction was performed. Of the 143 metabolites measured, 129 passed the QC check and were included in the final analysis. 16,512 pairwise metabolite ratios were calculated as a proxy for the enzymatic reaction and used in the analysis [17]. Linear regression models were used to identify metabolite ratio(s) that were associated with cartilage volume changes in each knee compartment with adjustment for age, sex, and BMI. The significance level of the association was defined as α = 0.05/16,512 = 3.03 × 10^−6^ with the Bonferroni method to control multiple testing. Confidence intervals of 95% (95% CI) were also provided. All analyses were performed in R version 3.4.3 with the built-in functions for linear regression. The visualization of the results was performed using the GraphPad Prism 9.1.0. (University of Rochester, Rochester, NY, USA).

## 3. Results

Of the 1099 participants recruited to the study at the baseline, a total of 563 completed follow-up at the average 10.8 ± 0.7 years. Among them, 476 participants had both baseline and follow-up MRI data, and 409 participants had serum metabolomics data. A total of 344 participants had both MRI and metabolomics data, and thereby were included in the current study.

The baseline characteristics of the participants and the changes in cartilage volume measured by the MRI scans are presented in Table 1. The rate of cartilage volume reduction in different compartments of the knee was different over the 10.8-year follow-up. The medial tibial cartilage had the largest annual reduction (−1.34 ± 0.72%), followed by the lateral tibial cartilage (−1.06 ± 0.58%). The patellar cartilage had the smallest annual reduction (−0.98 ± 0.46%).

**Figure 1:**
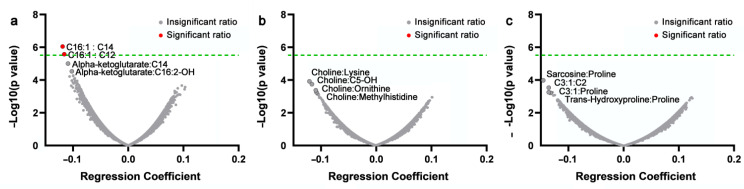
Volcano plot for the associations between metabolite ratios and cartilage volume changes. (**a**) Associations between all of the metabolite ratios and patellar cartilage volume changes over 10 years. (**b**) Associations between all of the metabolite ratios and lateral cartilage volume changes over 10 years. (**c**) Associations between all of the metabolite ratios and medial cartilage volume changes over 10 years. (Dash line indicates the pre-defined significance level) C16:1, hexadecenoylcarnitine; C14, tetradecanoylcarnitine; C12, dodecanoylcarnitine.

Among the 143 metabolites measured, 129 metabolites passed the quality control and were used for metabolite ratio calculation. A total of 16,512 pairwise metabolite ratios were generated and included in the subsequent analysis. Volcano plots (Figure 1a–c) present the results of the associations between each of the metabolite ratios and cartilage volume changes in each of the three compartments of the knee.

Our data showed that the ratios of hexadecenoylcarnitine (C16:1) to tetradecanoylcarnitine (C14) and dodecanoylcarnitine (C12) were significantly associated with the patellar cartilage volume change (*p* = 8.80 × 10^−7^ and 2.66 × 10^−6^, respectively) (Figure 1a) after adjustment for age, sex, and BMI. Per unit increase in each of the two ratios was associated with 0.12% reduction in the patellar cartilage volume per year (Table 2). Furthermore, although they did not reach the pre-defined significance level, the ratios of alpha-ketoglutarate to C14 and hydroxyhexadecadienylcarnitine (C16:2-OH) showed potential associations with patella cartilage volume loss (Table 2).

While we did not find any associations with medial and lateral cartilage volume loss with the pre-defined significance level (Figure 1b,c), the top four ratios with both medial and lateral cartilage volume changes are presented in Table 3 and Table 4. It appeared that choline was the key metabolite associated with lateral cartilage volume change (Table 3), whereas proline was the key metabolite associated with medial cartilage volume change, warranting further investigation.

## 4. Discussion

To the best of our knowledge, this was the first study to investigate the metabolic alterations in relation to the knee cartilage volume changes over 10 years in a large population-based cohort of older adults. In this study, we did not use individual serum metabolite concentrations but metabolite ratios for analysis. This was because the ratio of two interrelated metabolites as a proxy for the enzymatic reaction rate can more accurately reflect the development of the disease [17,18]. Moreover, studies have shown that the use of metabolite ratios in biomarker research can reduce the influence of confounding factors and produce more statistical power than single metabolites [19,20]. Furthermore, by using the cartilage volume loss rate assessed by MRI, we can more objectively and continuously detect the structural changes of joint tissues and then predict the progression of OA disease [21,22].

The results of this study showed that the serum concentration ratios of acylcarnitine C16:1 to acylcarnitine C14 and C12 could predict patella cartilage volume loss over 10 years. Acylcarnitine was first discovered more than 70 years ago [23], and it is believed that there are more than 1000 species of acylcarnitine in the human body. The general role of acylcarnitine is to transport acyl groups (organic acids and fatty acids) from the cytoplasm to the mitochondria for β-oxidation to produce energy [24]. Acylcarnitine can be divided into four categories based on the size of their acyl groups, including short-chain, medium-chain, long-chain, and very long-chain acylcarnitine. Acylcarnitine C16:1 and C14 are classified as long-chain acylcarnitine, and C12 could be classified as medium-chain acylcarnitine. Medium- and long-chain acylcarnitines are generally formed by the esterification of medium- and long-chain fatty acids. The main function of most long-chain acylcarnitines is to ensure the transport of long-chain fatty acids to mitochondria [25], participate in the β-oxidation of medium- and long-chain fatty acids, and continuously produce acylcarnitines with two carbon atoms less than the originals in the process of providing energy to the cell. Therefore, the acylcarnitines C14 and C12 could be the product of acylcarnitine C16:1 in the process of incomplete β-oxidation. The changes in the acylcarnitine ratios may indicate the metabolic dysfunction of the acylcarnitine involved in the β-oxidation of long-chain fatty acids. The availability of free carnitine might be another factor to explain the observed association. However, we did not find any association between any metabolite ratios involving free carnitine nor free carnitine itself with cartilage volume loss. Further, normalization of the ratios of C16:1 to C14 and to C12 by free carnitine did not alter the results, supporting the hypothesis that the incomplete β-oxidation of long-chain fatty acids rather than the availability of free carnitine is the most likely explanation of the observed association.

In the previous study of the synovial fluid samples of 80 end-stage OA patients who underwent total joint replacement surgery, our team demonstrated that two OA subtypes can be distinguished based on different synovial acylcarnitine concentrations and acetyltransferase activity [26]. Moreover, the study also found that OA subtypes with lower acylcarnitine concentrations have a higher prevalence of metabolic diseases and cardiovascular diseases. In support, a study by Tootsi et al. [27] also found that the serum levels of medium and long-chain acylcarnitines in OA patients were significantly lower than those in the control group and were correlated with the radiological severity of OA. Furthermore, the study showed that serum acylcarnitine level was an independent predictor of atherosclerosis. This suggested that acylcarnitine might play an important role in the link between OA and metabolic diseases or cardiovascular diseases, and the potential mechanism is likely related to the β-oxidation of fatty acid metabolism in mitochondria. In addition, studies have shown that acylcarnitine metabolism was significantly related to the progression of another arthritis disease, rheumatoid arthritis (RA) [28,29]. In the study conducted by Beyer et al. on the serum of 78 RA patients, they found that the changes in mitochondrial function related to the inflammatory state in RA disease may be related to the metabolism of acylcarnitine [28]. Andonian et al. showed in the study of 48 RA patients that the abnormal metabolism of long-chain acylcarnitine may be an internal link between RA inflammation and cardiometabolic comorbidities [29]. In support, abnormal acylcarnitine metabolism was reported to be highly related to many other diseases related to mitochondrial metabolic dysfunction, such as diabetes, metabolic syndrome, heart disease and obesity [30,31,32,33]. This might suggest that the main mechanism for abnormal acylcarnitine metabolism leading to the progression of OA disease is likely to be the same as in most aging-related diseases, namely the imbalance of cellular energy caused by mitochondrial dysfunction.

**Figure 2:**
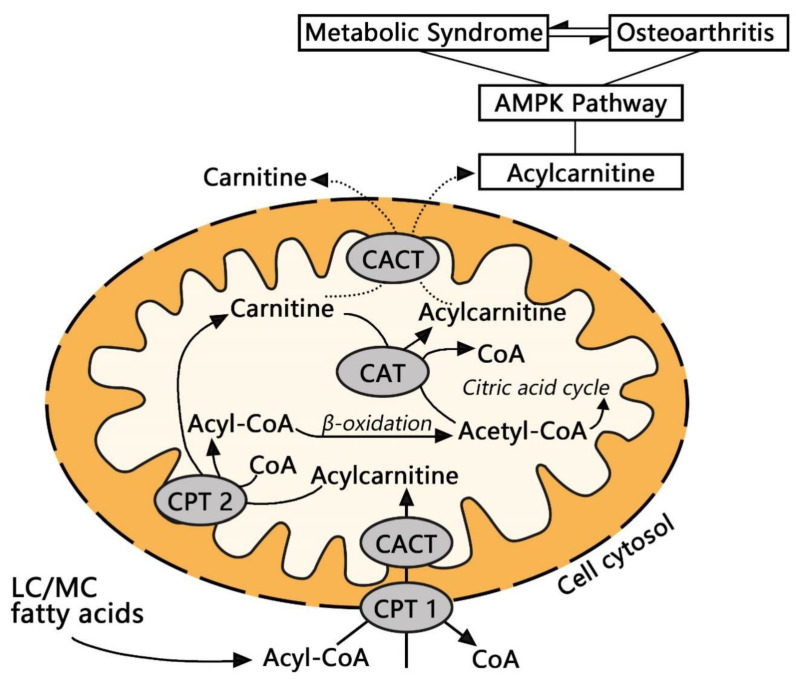
An overview of fatty acid oxidation, carnitine biosynthesis and metabolism, and AMPK pathway. CPT, carnitine palmitoyl-transferase; CACT, carnitine-acylcarnitine translocase; CAT, Carnitine Acyltransferase; LC/MC, long-chain/medium-chain; AMPK, adenosine monophosphate activated protein kinase; CoA, Coenzyme A.

Therefore, metabolic syndrome, as the most common comorbidity of OA patients, is worthy of our attention. According to data from the NHANES III cohort [34], approximately half of American OA patients also have metabolic syndrome. Cross-sectional studies based on other populations have also found that there is a connection between metabolic syndrome and knee OA, and the two promote each other in the occurrence and development of the disease [35]. Recently emerging evidence suggested that metabolic abnormalities play a key role in the pathogenesis of OA, which can also be demonstrated by the increased incidence and severity of OA in patients with metabolic syndrome such as obesity, insulin resistance, and hyperlipidemia [36]. Interestingly, it is well known that as a key bioenergy sensor, AMP-activated protein kinase (AMPK) not only mediates energy homeostasis, but also mediates the redox balance of chondrocytes to resist various cellular stresses. Abnormal AMPK activity is related to decreased autophagy, impaired mitochondrial function, excessive production of reactive oxygen species, and inflammation of joint tissues [37]. Previous studies have found that the imbalance of AMPK activity in chronic diseases can cause mitochondrial dysfunction and acylcarnitine metabolism imbalance [38]. Studies have also shown that the abnormal AMPK activity plays an important role in the association between OA and metabolic syndrome [39]. This suggests that cartilage destruction in the progress of OA may be caused by the abnormal activation of the AMPK pathway and the imbalance of the acylcarnitine metabolism (Figure 2). However, we currently do not have relevant data on patients with OA to determine the correlation between abnormal acylcarnitine metabolism, mitochondrial metabolism disorder and AMPK activity imbalance, which needs to be tested in future studies.

It is not clear why the association between the ratio of acylcarnitine and the rate of the patellar cartilage volume change was more significant than that of the other two compartments. As we know, there are many patients with OA in the moderate to severe stage that lose patella cartilage at a faster rate than they lose cartilage in the other two compartments. By using a very conservative method to control for multiple comparisons, we did not find any significant association between the metabolite ratios and the volume loss of the medial and lateral cartilage. However, three of the four metabolite ratios with the highest association with medial cartilage reduction were related to proline. This suggests that cartilage loss in patients with OA may be related to proline metabolism. In line with this, previous studies have found that proline plays a protective role in the progression of OA disease and helps the regeneration of articular cartilage [40]. In contrast, all of the four metabolite ratios with the highest correlation with lateral cartilage reduction were related to choline metabolism. Previous studies have found that choline plays a role in the transmission of pain information in the progression of OA disease and controls the oxidative stress of chondrocytes [41]. Thus, we could speculate that choline metabolism also plays a role in the process of articular cartilage loss.

Although this study has several advantages, including longitudinal study design, long-term follow-up, community-based sampling and MRI detection structure, there were some potential limitations that need to be recognized when interpreting the findings. First, not all participants included in the study had OA, which means that the data obtained by this analysis may not accurately represent the cartilage loss of the OA patient population. Secondly, because of the metabolite detection method, we cannot distinguish the structural details between the measured compounds of the same mass. This needs to be improved with higher resolution methods in the future. Thirdly, the cohort was selected from the general population and we don’t have data on chronic diseases such as chronic inflammatory disease, dyslipidemia, atherosclerosis, and rheumatoid arthritis, which might be potential confounders. Fourthly, we don’t have knee OA incidence data, which prevented us from assessing whether the ratios are associated with the development of knee OA or not. Fifth, we currently do not have enough data to analyze the relationship between abnormal acylcarnitine metabolism and mitochondrial dysfunction and the imbalance of AMPK and other biological enzyme activities in cartilage loss of OA patients. Lastly, the study only included one cohort, and further validation from an independent cohort is needed.

## 5. Conclusions

In summary, our data suggest that the alteration in fatty acid β-oxidation is related to the loss of knee joint cartilage, especially in the patella compartment. The serum ratios of acylcarnitine C16:1 to C14 and C12 might be used as new biomarkers for predicting long-term patellar cartilage loss.

## Figures and Tables

**Table 1:** Descriptive characteristics of the study population.

Variables	Participants	Mean ± SD	Max	Min
Age (years)	*n* = 344	62.83 ± 6.13	82.10	53.20
Sex (% female)	*n* = 344	51.00		
BMI (kg/m^2^)	*n* = 344	27.48 ± 4.41	47.34	18.77
Follow-up time (years)	*n* = 344	10.84 ± 0.66	12.59	9.35
Lateral cartilage volume at baseline (mm^3^)	*n* = 344	2073.10 ± 600.21	3686.90	919.20
Lateral cartilage volume change per year (%)	*n* = 344	−1.06 ± 0.58	−4.27	0.22
Medial cartilage volume at baseline (mm^3^)	*n* = 344	1511.78 ± 413.24	3037.90	745.60
Medial cartilage volume change per year (%)	*n* = 344	−1.34 ± 0.72	−5.68	0.37
Patellar cartilage volume at baseline (mm^3^)	*n* = 343	2540.40 ± 703.37	4607.00	966.80
Patellar cartilage volume change per year (%)	*n* = 343	−0.98 ± 0.46	−3.81	0.01

Results are shown as mean ± SD except for sex, which was shown as a percentage for females. BMI, body mass index.

**Table 2:** Top four metabolite ratios associated with the percentage of patellar cartilage volume loss per year.

Metabolite Ratios	β (95% CI)	*p* Value
C16:1:C14	−0.12 (−0.10, −0.14)	8.80 × 10^−7^
C16:1:C12	−0.12 (−0.10, −0.14)	2.66 × 10^−6^
Alpha-ketoglutarate:C14	−0.11 (−0.09, −0.13)	9.97 × 10^−6^
Alpha-ketoglutarate:C16:2-OH	−0.10 (−0.08, −0.12)	2.90 × 10^−5^

Associations were tested by using the linear regression model. Covariates including age, sex and body mass index were included in the multivariable linear regression model. C16:1, hexadecenoylcarnitine; C14, tetradecanoylcarnitine; C12, dodecanoylcarnitine; C16:2-OH, hydroxyhexadecadienylcarnitine.

**Table 3:** Top four metabolite ratios associated with the percentage of lateral cartilage volume loss per year.

Metabolite Ratios	β (95% CI)	*p* Value
Choline:Lysine	−0.12 (−0.09, −0.15)	1.18 × 10^−4^
Choline:C5-OH	−0.12 (−0.09, −0.15)	1.75 × 10^−4^
Choline:Ornithine	−0.11 (−0.08, −0.14)	4.43 × 10^−4^
Choline:Methylhistidine	−0.11 (−0.08, −0.14)	4.80 × 10^−4^

Associations were tested by using a linear regression model. Covariates including age, sex and body mass index were included in the multivariable linear regression model. C5-OH, hydroxyvalerylcarnitine.

**Table 4:** Top four metabolite ratios associated with the percentage of medial cartilage volume loss per year.

Metabolite Ratios	β (95% CI)	*p* Value
Sarcosine:Proline	−0.15 (−0.11, −0.19)	1.03 × 10^−4^
C3:1:C2	−0.14 (−0.10, −0.18)	2.91 × 10^−4^
C3:1:Proline	−0.13 (−0.09, −0.17)	5.39 × 10^−4^
Trans-Hydroxyproline:Proline	−0.13 (−0.09, −0.17)	5.41 × 10^−4^

Associations were tested by using a linear regression model. Covariates including age, sex and body mass index were included in the multivariable linear regression model. C3:1, propenoylcarnitine; C2, acetylcarnitine.

## Data Availability

The datasets used and/or analyzed during the current study are available from the corresponding author on reasonable request.

## Supplementary Materials

The following supporting information can be downloaded at: https://www.mdpi.com/article/10.3390/life12060869/s1, Table S1: List of metabolites measured by the TMIC Primer Metabolomics Profiling Assay.

## Abbreviations

AMPK	Adenosine Monophosphate Activated Protein Kinase;
BMI	Body Mass Index;
CV	Coefficient of Variation;
CI	Confidence Intervals;
C16:1	Hexadecenoylcarnitine;
C14	Tetradecanoylcarnitine;
C12	Dodecanoylcarnitine;
C5-OH	Hydroxyvalerylcarnitine;
C16:2-OH	Hydroxyhexadecadienylcarnitine;
C3:1	Propenoylcarnitine;
C2	Acetylcarnitine;
CPT	Carnitine Palmitoyl-transferase;
CACT	Carnitine-acylcarnitine Translocase;
CAT	Carnitine Acyltransferase;
CoA	Coenzyme A;
lysoPC 18:2	Lysophosphatidylcholine 18:2;
LOD	Limit of Detection;
LC/MC	Long-chain/Medium-chain;
MRI	Magnetic Resonance Imaging;
NHANES III	The Third National Health and Nutrition Examination Survey;
OA	Osteoarthritis;
PC 44:3	Phosphatidylcholine 44:3;
PCA	Principal component analysis;
QC	Quality Control;
RA	Rheumatoid Arthritis;
TASOAC	Tasmania Older Adult Cohort;
TMIC	The Metabolomics Innovation Centre;
UHPLC	Ultra-high-performance Liquid Chromatography.

## References

[1] WHO Scientific Group on the Burden of Musculoskeletal Conditions at the Start of the New Millennium (2003). The burden of musculoskeletal conditions at the start of the new millennium. World Health Organ. Tech. Rep. Ser..

[2] Hiligsmann M., Cooper C., Arden N., Boers M., Branco J.C., Luisa Brandi M., Bruyère O., Guillemin F., Hochberg M.C., Hunter D.J. (2013). Health economics in the field of osteoarthritis: An expert’s consensus paper from the European Society for Clinical and Economic Aspects of Osteoporosis and Osteoarthritis (ESCEO). Semin. Arthritis Rheum..

[3] Bingham C.O., Buckland-Wright J.C., Garnero P., Cohen S.B., Dougados M., Adami S., Clauw D.J., Spector T.D., Pelletier J.P., Raynauld J.P. (2006). Risedronate decreases biochemical markers of cartilage degradation but does not decrease symptoms or slow radiographic progression in patients with medial compartment osteoarthritis of the knee: Results of the two-year multinational knee osteoarthritis structural arthritis study. Arthritis Rheum..

[4] Cai G., Jiang M., Cicuttini F., Jones G. (2019). Association of age, sex and BMI with the rate of change in tibial cartilage volume: A 10.7-year longitudinal cohort study. Arthritis Res. Ther..

[5] Raynauld J.P., Martel-Pelletier J., Bias P., Laufer S., Haraoui B., Choquette D., Beaulieu A.D., Abram F., Dorais M., Vignon E. (2009). Protective effects of licofelone, a 5-lipoxygenase and cyclo-oxygenase inhibitor, versus naproxen on cartilage loss in knee osteoarthritis: A first multicentre clinical trial using quantitative MRI. Ann. Rheum. Dis..

[6] Zhai G., Randell E.W., Rahman P. (2018). Metabolomics of osteoarthritis: Emerging novel markers and their potential clinical utility. Rheumatology.

[7] Zhai G. (2021). The role of metabolomics in precision medicine of osteoarthritis: How far are we?. Osteoarthr. Cartil. Open.

[8] Carlson A.K., Rawle R.A., Adams E., Greenwood M.C., Bothner B., June R.K. (2018). Application of global metabolomic profiling of synovial fluid for osteoarthritis biomarkers. Biochem. Biophys. Res. Commun..

[9] Xu Z., Chen T., Luo J., Ding S., Gao S., Zhang J. (2017). Cartilaginous Metabolomic Study Reveals Potential Mechanisms of Osteophyte Formation in Osteoarthritis. J. Proteome Res..

[10] Tootsi K., Vilba K., Märtson A., Kals J., Paapstel K., Zilmer M. (2020). Metabolomic Signature of Amino Acids, Biogenic Amines and Lipids in Blood Serum of Patients with Severe Osteoarthritis. Metabolites.

[11] Zhang W., Sun G., Aitken D., Likhodii S., Liu M., Martin G., Furey A., Randell E., Rahman P., Jones G. (2016). Lysophosphatidylcholines to phosphatidylcholines ratio predicts advanced knee osteoarthritis. Rheumatology.

[12] Zhai G., Pelletier J.P., Liu M., Aitken D., Randell E., Rahman P., Jones G., Martel-Pelletier J. (2019). Activation of The Phosphatidylcholine to Lysophosphatidylcholine Pathway Is Associated with Osteoarthritis Knee Cartilage Volume Loss Over Time. Sci. Rep..

[13] Zhai G., Cicuttini F., Srikanth V., Cooley H., Ding C., Jones G. (2005). Factors associated with hip cartilage volume measured by magnetic resonance imaging: The Tasmanian Older Adult Cohort Study. Arthritis Rheum..

[14] Munugoda I.P., Pan F., Wills K., Mattap S.M., Cicuttini F., Graves S.E., Lorimer M., Jones G., Callisaya M.L., Aitken D. (2020). Identifying subgroups of community-dwelling older adults and their prospective associations with long-term knee osteoarthritis outcomes. Clin. Rheumatol..

[15] Jones G., Glisson M., Hynes K., Cicuttini F. (2000). Sex and site differences in cartilage development: A possible explanation for variations in knee osteoarthritis in later life. Arthritis Rheum..

[16] Mittelstrass K., Ried J.S., Yu Z., Krumsiek J., Gieger C., Prehn C., Roemisch-Margl W., Polonikov A., Peters A., Theis F.J. (2011). Discovery of sexual dimorphisms in metabolic and genetic biomarkers. PLoS Genet..

[17] Zhang W., Likhodii S., Aref-Eshghi E., Zhang Y., Harper P.E., Randell E., Green R., Martin G., Furey A., Sun G. (2015). Relationship between blood plasma and synovial fluid metabolite concentrations in patients with osteoarthritis. J. Rheumatol..

[18] Petersen A.K., Krumsiek J., Wägele B., Theis F.J., Wichmann H.E., Gieger C., Suhre K. (2012). On the hypothesis-free testing of metabolite ratios in genome-wide and metabolome-wide association studies. BMC Bioinform..

[19] Molnos S., Wahl S., Haid M., Eekhoff E., Pool R., Floegel A., Deelen J., Much D., Prehn C., Breier M. (2018). Metabolite ratios as potential biomarkers for type 2 diabetes: A DIRECT study. Diabetologia.

[20] Heemskerk M.M., van Harmelen V.J., van Dijk K.W., van Klinken J.B. (2016). Reanalysis of mGWAS results and in vitro validation show that lactate dehydrogenase interacts with branched-chain amino acid metabolism. Eur. J. Hum. Genet..

[21] Pelletier J.P., Cooper C., Peterfy C., Reginster J.Y., Brandi M.L., Bruyère O., Chapurlat R., Cicuttini F., Conaghan P.G., Doherty M. (2013). What is the predictive value of MRI for the occurrence of knee replacement surgery in knee osteoarthritis?. Ann Rheum Dis..

[22] Nguyen H.H., Wu F., Oddy W.H., Wills K., Brennan-Olsen S.L., Jones G., Winzenberg T. (2021). Longitudinal associations of dietary patterns with sociodemographic and lifestyle factors in older adults: The TASOAC study. Eur. J. Clin. Nutr..

[23] Fritz I.B. (1959). Action of carnitine on long chain fatty acid oxidation by liver. Am. J. Physiol..

[24] Indiveri C., Iacobazzi V., Tonazzi A., Giangregorio N., Infantino V., Convertini P., Console L., Palmieri F. (2011). The mitochondrial carnitine/acylcarnitine carrier: Function, structure and physiopathology. Mol. Asp. Med..

[25] Reuter S.E., Evans A.M. (2012). Carnitine and acylcarnitines: Pharmacokinetic, pharmacological and clinical aspects. Clin. Pharmacokinet..

[26] Zhang W., Likhodii S., Zhang Y., Aref-Eshghi E., Harper P.E., Randell E., Green R., Martin G., Furey A., Sun G. (2014). Classification of osteoarthritis phenotypes by metabolomics analysis. BMJ Open.

[27] Tootsi K., Kals J., Zilmer M., Paapstel K., Ottas A., Märtson A. (2018). Medium- and long-chain acylcarnitines are associated with osteoarthritis severity and arterial stiffness in end-stage osteoarthritis patients: A case-control study. Int. J. Rheum. Dis..

[28] Beyer K., Lie S.A., Bjørndal B., Berge R.K., Svardal A., Brun J.G., Bolstad A.I. (2021). Lipid, fatty acid, carnitine- and choline derivative profiles in rheumatoid arthritis outpatients with different degrees of periodontal inflammation. Sci. Rep..

[29] Andonian B.J., Chou C.H., Ilkayeva O.R., Koves T.R., Connelly M.A., Kraus W.E., Kraus V.B., Huffman K.M. (2019). Plasma MicroRNAs in Established Rheumatoid Arthritis Relate to Adiposity and Altered Plasma and Skeletal Muscle Cytokine and Metabolic Profiles. Front. Immunol..

[30] Wahl S., Yu Z., Kleber M., Singmann P., Holzapfel C., He Y., Mittelstrass K., Polonikov A., Prehn C., Römisch-Margl W. (2012). Childhood obesity is associated with changes in the serum metabolite profile. Obes. Facts.

[31] Schooneman M.G., Vaz F.M., Houten S.M., Soeters M.R. (2013). Acylcarnitines: Reflecting or inflicting insulin resistance?. Diabetes.

[32] Bruce C.R., Hoy A.J., Turner N., Watt M.J., Allen T.L., Carpenter K., Cooney G.J., Febbraio M.A., Kraegen E.W. (2009). Overexpression of carnitine palmitoyltransferase-1 in skeletal muscle is sufficient to enhance fatty acid oxidation and improve high-fat diet-induced insulin resistance. Diabetes.

[33] Ahmad T., Kelly J.P., McGarrah R.W., Hellkamp A.S., Fiuzat M., Testani J.M., Wang T.S., Verma A., Samsky M.D., Donahue M.P. (2016). Prognostic Implications of Long-Chain Acylcarnitines in Heart Failure and Reversibility with Mechanical Circulatory Support. J. Am. Coll. Cardiol..

[34] Puenpatom R.A., Victor T.W. (2009). Increased prevalence of metabolic syndrome in individuals with osteoarthritis: An analysis of NHANES III data. Postgrad. Med..

[35] Lee S., Kim T.N., Kim S.H., Kim Y.G., Lee C.K., Moon H.B., Koh E.M., Yoo B. (2015). Obesity, metabolic abnormality, and knee osteoarthritis: A cross-sectional study in Korean women. Mod. Rheumatol..

[36] June R.K., Liu-Bryan R., Long F., Griffin T.M. (2016). Emerging role of metabolic signaling in synovial joint remodeling and osteoarthritis. J. Orthop. Res..

[37] Wang J., Li J., Song D., Ni J., Ding M., Huang J., Yan M. (2020). AMPK: Implications in osteoarthritis and therapeutic targets. Am. J. Transl. Res..

[38] Koh J.H., Kim K.H., Park S.Y., Kim Y.W., Kim J.Y. (2020). PPARδ Attenuates Alcohol-Mediated Insulin Resistance by Enhancing Fatty Acid-Induced Mitochondrial Uncoupling and Antioxidant Defense in Skeletal Muscle. Front. Physiol..

[39] Song P., Hwang J.S., Park H.C., Kim K.K., Son H.J., Kim Y.J., Lee K.M. (2021). Therapeutic Applications of Type 2 Diabetes Mellitus Drug Metformin in Patients with Osteoarthritis. Pharmaceuticals.

[40] Li M., Xiao Y.B., Wang X.T., Zhuang J.P., Zhou C.L. (2021). Proline-Serine-Threonine Phosphatase-Interacting Protein 2 Alleviates Diabetes Mellitus-Osteoarthritis in Rats through Attenuating Synovial Inflammation and Cartilage Injury. Orthop. Surg..

[41] Beckmann J., Schubert J., Morhenn H.G., Grau V., Schnettler R., Lips K.S. (2015). Expression of choline and acetylcholine transporters in synovial tissue and cartilage of patients with rheumatoid arthritis and osteoarthritis. Cell Tissue Res..

