# Supplementary material for: Serum Metabolomic Signatures for Knee Cartilage Volume Loss over 10 Years in Community-Dwelling Older Adults
Source: Life (Basel). 2022 Jun 10;12(6):869. doi: 10.3390/life12060869 (PMC9225196; doi:10.3390/life12060869)
Supplement: Supplementary file 1 [file life-12-00869-s001.zip › life-1732793-supplementary.pdf]

Supplementary Table S1: List of metabolites measured by the TMIC Prime Metabolomics Profiling Assay

Metabolite class	Number	Metabolite name or abbreviation
Carnitine	1	C0
Acylcarnitines	25	C2, C3, C3:1, C4, C4:1, C5, C5:1, C6(or C4:1-DC), C6:1, C8, C9, C10, C10:1, C10:2, C12, C12:1, C14, C14:1, C14:2, C16, C16:1, C16:2, C18, C18:1, C18:2
Hydroxy- and dicarboxyacylcarnitines	14	C3-OH, C4-OH(or C3-DC), C5:1-DC, C5-DC(or C6-OH), C5-M-DC, C5-OH(or C3-DC-M), C7-DC, C12-DC, C14:1-OH, C14:2-OH, C16:1-OH, C16:2-OH, C16-OH, C18:1-OH
Amino acids and derivatives	25	Alanine, arginine, asparagine, aspartate, citrulline, glutamine, glutamate, glycine, histidine, isoleucine, leucine, lysine, methionine, ornithine, phenylalanine, proline, serine, threonine, tryptophan, tyrosine, valine, betaine, creatine, phosphocreatine, methylhistidine
Amine oxides	1	Trimethylamine N-oxide (TMAO)
Biogenic amines	23	Acetylornithine, asymmetric dimethylarginine, total dimethylarginine, alpha-aminoadipic acid, carnosine, creatinine, dopa, dopamine, histamine, kynurenine, methionine sulfoxide, hydroxyproline (t4-OH-Pro), hydroxyproline (c4-OH-Pro), phenylethylamine, putrescine, sarcosine, serotonin, spermidine, spermine, diacetylspermine, taurine, tyramine, nitrotyrosine
Carboxylic acids	1	Homocysteine
Monosaccharides	1	Glucose
Organic acids	17	Lactic acid, beta-hydroxybutyric acid, alpha-ketoglutaric acid, citric acid, butyric acid, propionic acid, 3-(3-hydroxyphenyl)-3-hydroxypropionic acid (HPHPA), para-hydroxyhippuric acid, succinic acid, fumaric acid, pyruvic acid, isobutyric acid, hippuric acid, methylmalonic acid, homovanillic acid, indole acetic acid, uric acid
Lyso-phosphatidylcholines	14	LysoPC a C14:0/C16:0/C16:1/C17:0/C18:0/C18:1/C18:2/ C20:3/C20:4/C24:0/C26:0/C26:1/C28:0/C28:1
Diacyl-phosphatidylcholines	8	PC aa C32:2/C36:0/C36:6/C38:0/C38:6/C40:1/C40:2/C40:6
Acyl-alkyl-phosphatidylcholines	2	PC ae C36:0/C40:6
Sphingomyelines	5	SM C16:0, SM C16:1, SM C18:0, SM C18:1, SM C20:2
Hydroxysphingomyelines	5	SM (OH) C14:1, SM (OH) C16:1, SM (OH) C22:1, SM (OH) C22:2, SM (OH) C24:1
Vitamins and cofactors	1	Choline
Total	143	

aa: acyl-acyl; ae: acyl-alkyl; a: acyl; Cx:y: where x is the number of carbons in the fatty acid side chain; y is the number of double bonds in the fatty acid side chain; DC: decarboxyl; M: methyl; OH: hydroxyl; PC: phophatidylcholine; SM: sphingomyelin.
